# Characterizing the molecular heterogeneity of clear cell renal cell carcinoma subgroups classified by miRNA expression profile

**DOI:** 10.3389/fmolb.2022.967934

**Published:** 2022-08-26

**Authors:** Tao Shen, Yingdong Song, Xiangting Wang, Haiyang Wang

**Affiliations:** ^1^ Anhui Provincial Key Laboratory of Molecular Enzymology and Mechanism of Major Diseases, Key Laboratory of Biomedicine in Gene Diseases, Health of Anhui Higher Education Institutes, College of Life Sciences, Anhui Normal University, Wuhu, China; ^2^ Hefei National Research Center for Physical Sciences at the Microscale, University of Science and Technology of China, Hefei, China; ^3^ Department of Geriatrics, Gerontology Institute of Anhui Province, The First Affiliated Hospital, Division of Life Sciences and Medicine, University of Science and Technology of China, Hefei, China; ^4^ Anhui Provincial Key Laboratory of Tumor Immunotherapy and Nutrition Therapy, Hefei, China

**Keywords:** microRNA, consensus molecular subtypes, clear cell renal cell carcinoma, miRNA-regulated networks, transmembrane transport activity

## Abstract

Clear cell renal cell carcinoma (ccRCC) is a heterogeneous disease that is associated with poor prognosis. Recent works have revealed the significant roles of miRNA in ccRCC initiation and progression. Comprehensive characterization of ccRCC based on the prognostic miRNAs would contribute to clinicians’ early detection and targeted treatment. Here, we performed unsupervised clustering using TCGA-retrieved prognostic miRNAs expression profiles. Two ccRCC subtypes were identified after assessing principal component analysis (PCA), t-distributed stochastic neighbor embedding (t-SNE), and consensus heatmaps. We found that the two subtypes are associated with distinct clinical features, overall survivals, and molecular characteristics. C1 cluster enriched patients in relatively early stage and have better prognosis while patients in C2 cluster have poor prognosis with relatively advanced state. Mechanistically, we found the differentially expressed genes (DEGs) between the indicated subgroups dominantly enriched in biological processes related to transmembrane transport activity. In addition, we also revealed a miRNA-centered DEGs regulatory network, which severed as essential regulators in both transmembrane transport activity control and ccRCC progression. Together, our work described the molecular heterogeneity among ccRCC cancers, provided potential targets served as effective biomarkers for ccRCC diagnosis and prognosis, and paved avenues to better understand miRNA-directed regulatory network in ccRCC progression.

## Introduction

Renal cell carcinoma (RCC) is among the top ten most commonly diagnosed cancers worldwide (Sung et al., 2021). Clear cell renal cell carcinoma (ccRCC) is the predominant histology type of RCC, representing 70–80% RCC cases with an estimation of 360,000 new cases and 150,000 deaths worldwide in 2020 (Sung et al., 2021). Although ccRCC is potentially curable by surgical or ablative strategies in its early stage, up to a third of cases diagnosed in advanced stages with or develop metastases due to the lack of clinical manifestations in its early stages. In contrast to patients in early stages, the prognosis for patients with the advanced ccRCC state is poor (Jonasch et al., 2021). Targeted therapy is currently the first-line treatment for such cases. This involves the use of tyrosine kinase inhibitors (TKIs), TOR inhibitors, monoclonal antibodies to vascular endothelial growth factor (VEGF) and immune checkpoint inhibitors (ICIs) (Braga et al., 2021; Mori et al., 2021). Unfortunately, not all patients are susceptible to those therapy, and over time, the targeted therapy and the use of checkpoint inhibitors can develop resistance (Jenkins et al., 2018; Rosenzweig 2018). Therefore, it is urgent to discover novel level of biomarkers for early diagnosis and provide potential targets to facilitate the efficiency of targeted therapy.

One of the recently discovered levels of regulation is the action of microRNAs (miRNAs), which are a class of small endogenous non-coding RNA of 19–22 nucleotides (Marchioni et al., 2021; Tito et al., 2021). Through regulated numerous targeted functional gene products, miRNAs are involved in many aspects of cancer development (Mendell and Olson 2012). Recent literatures reported that the alteration of miRNA was closely linked with ccRCC tumorigenesis and recurrence and highlighted that these miRNAs expression level in ccRCC tissue provide diagnostic and prognostic information (Hildebrandt et al., 2010; Gebauer et al., 2013; Wotschofsky et al., 2013; Fu et al., 2014; Samaan et al., 2015; Shu et al., 2017; Zhang et al., 2018; Saleeb et al., 2019). In addition, miRNAs are also circulating in blood and they are characterized by a remarkable stability against degradation by RNases, pH changes, and freeze/thawing, which makes miRNA became an important matter for biomarker researchers (Chen et al., 2008; Heinemann et al., 2018).

Given the important role of miRNA in ccRCC diagnosis and prognosis, we systematically investigated the molecular heterogeneity of ccRCC based on prognosis-related miRNA expression in the present study. We identified two subtypes with distinct molecular and clinical characteristics and revealed the potential regulatory network responsible for the differences. These results would lay a foundation for better understanding of ccRCC’s pathogenesis and provide alternative choices for early diagnosis and targeted therapy.

## Materials and methods

### Data download and preprocessing

The miRNA-sequence data (isoform expression quantification) were obtained from the Cancer Genome Atlas (TCGA, https://portal.gdc.cancer.gov/) database. Firstly, the miRNAs with missing values of more than 20% in all samples were removed. Subsequently, we retrieved the information of mature miRNA corresponding to the miMAT accession numbr using the R package miRBaseVersions.db (Haunsberger, Connolly, and Prehn 2017). The RNA-sequence data (fragments per kilobase of exon model per million mapped, FPKM), copy number variation (gene-level), clinical information and phenotype information of ccRCC patients were downloaded from the UCSC Xena website (http://xena.ucsc.edu/). The miRNA-array data and the related clinical information were obtained from GSE131959 in Gene Expression Omnibus (GEO, https://www.ncbi.nlm.nih.gov/geo/) database. The RNA-sequence data of RCC patients were downloaded from the ICGC (https://dcc.icgc.org/) database.

### Survival analysis and subtyping

Univariate Cox regression analysis was used to identify prognosis-related miRNAs. Variables with *p* value <0.01 in univariate Cox were further used for multivariate Cox regression analysis to determine whether they could function as independent prognostic factors along with the clinical factors (including age, gender, tumor stage and tumor grade). The hazard ratios (HRs) with 95% confidence intervals (CI) and log-rank *p* values were also computed. These results were obtained from the survival R package and visualized by the ggplot2 R package.

Consensus clustering (the “ConsensusClusterPlus” package in R) (Wilkerson and Hayes 2010) was performed to determine the optimal number of which independent prognosis related miRNAs based ccRCC subtypes. The principal component analysis (PCA) and t-distributed stochastic neighbor embedding (t-SNE) analysis (the “Rtsne” package in R) (Van Der Maaten 2014) were applied to verify the classification between Cluster 1 and Cluster 2. Then Kaplan–Meier survival analysis was performed to estimate the survival difference between these two clusters by using the survival and survminer R packages.

### Clinical and molecular characteristics identification of the indicated subgroups

The proportions of different clinical factors in the subgroups of ccRCC were statistically analyzed and visualized by ggplot2 R package. The differentially expressed genes (DEGs) between the two clusters were screened out by the limma R package (Schober, Boer, and Schwarte 2018) with the criteria of |log2 (fold change)| > 1 and adjusted *p* value <0.05.

### Functional enrichment analysis

The Gene Ontology (GO) was performed by limma and clusterProfiler R packages (Yu et al., 2012). GOplot R package was used to display the result of the functional enrichment analysis.

### Targets prediction of the DEipr-miRNAs

The differential expressed ipr-miRNAs (DEipr-miRNAs) between the two subgroups were screened out by the limma R package with the criteria of |log2 (fold change)| > 1 and adjusted *p* value <0.05. The target genes of DEipr-miRNAs were predicted using TargetScan (http://www.targetscan.org/), miRDB (http://www.mirdb.org/) and miRWalk (http://mirwalk.umm.uni-heidelberg.de/). The overlapped target genes were obtained by using the Venn diagram by the venn R package. According to DEGs between the two subgroups, we constructed regulatory networks of the DEipr-miRNAs and their targeted DEGs by using Cytoscape software (Saito et al., 2012).

### Identification of the prognostic DEipr-miRNAs-regulated DEGs

Firstly, the subcellular localization of DEipr-miRNAs-targeted DEGs’ mRNA were searched and recorded in RNALocate database (https://www.rna-society.org/rnalocate/). Then, we conduct statistical analysis of the subcellular distribution. According to the canonical function of miRNAs in cytoplasm, we focused on the cytosolic DEGs and screened out the DEipr-miRNAs-targeted DEGs which were expressed in an opposite direction to DEipr-miRNAs as the DEipr-miRNAs-regulated DEGs. Finally, univariate and multivariate cox regression analyses (*p* value <0.01) were used to determine the prognosis of DEipr-miRNAs-regulated DEGs in ccRCC patients.

### Expression, subcellular localization, and clinical validation of DEipr-miRNAs-regulated ipr-DEGs

The copy number variation (CNV) data were downloaded from UCSC Xena database to calculate the percentage of the indicated DEipr-miRNAs-regulated ipr-DEGs gain and loss in the total number of ccRCC patients, which indicated the gene level changes of the aforementioned ipr-DEGs. The RNA-sequence data were downloaded from TCGA and ICGC. Differential expression analysis was conducted and the screening standard (|log2 (fold change)| > 1 and adjusted *p* value <0.05) were set to validate the expression of the indicated DEipr-miRNAs-regulated ipr-DEGs in ccRCC and RCC normal and tumor samples in RNA level. The immunohistochemical data of the indicated DEipr-miRNAs-regulated ipr-DEGs were downloaded from the Human Protein Atlas database (HPA, https://www.proteinatlas.org/) (Digre and Lindskog 2021) to determine their expression in protein level. The immunofluorescence data of the indicated DEipr-miRNAs-regulated ipr-DEGs were downloaded from HPA database to represent their subcellular localization. Kaplan Meier-plotter method was performed to validate the relationship between the indicated DEipr-miRNAs-regulated ipr-DEGs and overall survival (OS) of TCGA-retrieved ccRCC patients.

## Results

### Identification of the independent prognosis-related miRNAs in clear cell renal cell carcinoma

**FIGURE 1:**
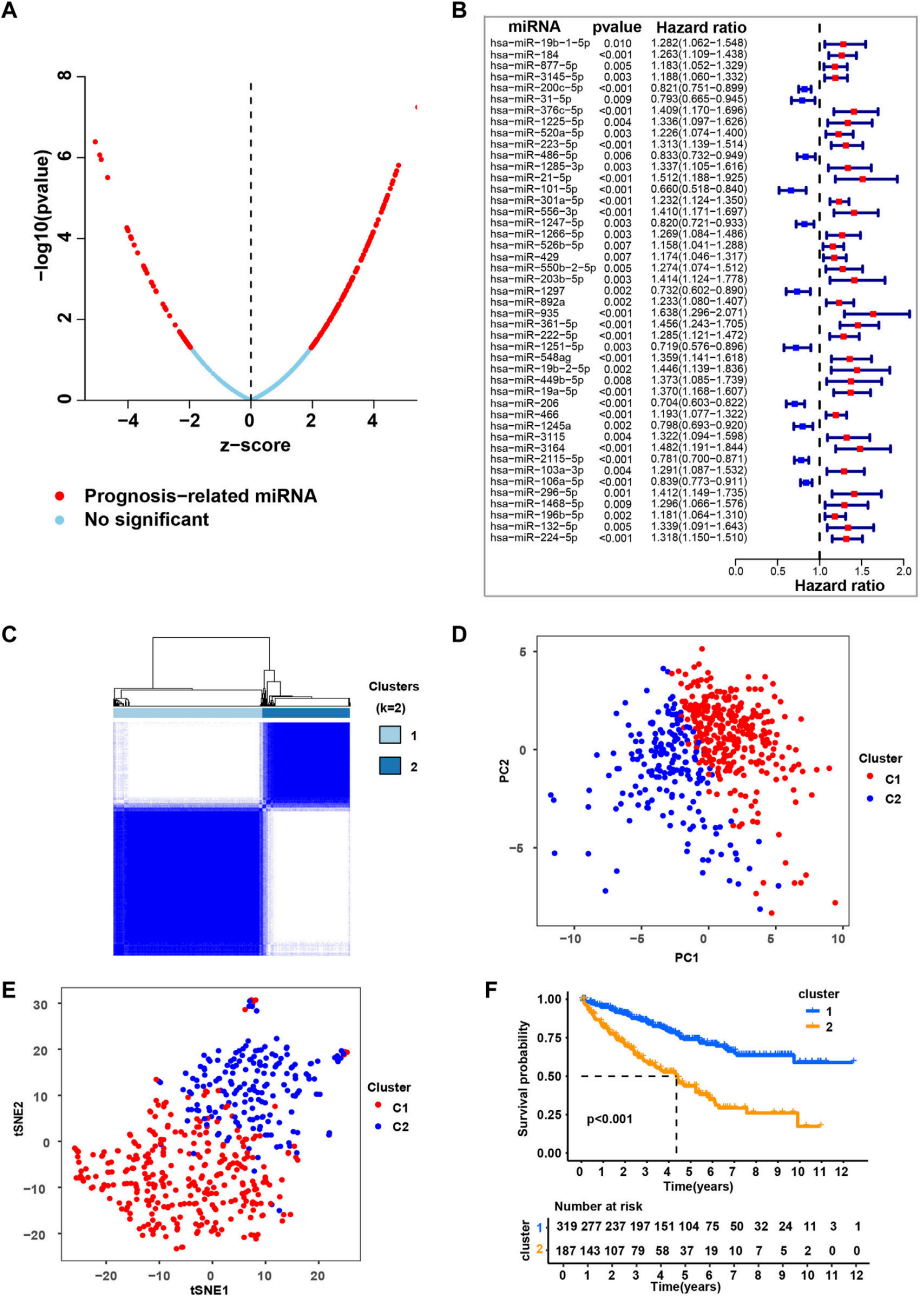
Consensus clustering based on the independent prognosis-related miRNAs for ccRCC. **(A)** Univariate cox regression analysis to identify the prognosis-related miRNAs (pr-miRNAs) in ccRCC. **(B)** Multivariate cox regression analysis to identify the independent prognosis-related miRNAs (ipr-miRNAs) in ccRCC. **(C)** Consensus matrix heatmap when k = 2. Related to Supplementary Figure S2. **(D)** Principal component analysis (PCA) for the TCGA-retrieved ccRCC patients, each dot represents a single sample. **(E)** T-distributed stochastic neighbor embedding (t-SNE) analysis for the TCGA-retrieved ccRCC patients, each dot represents a single sample. **(F)** Kaplan-Meier plot analysis for the indicated TCGA-retrieved ccRCC patients distributed in Cluster1 (C1) and Cluster2 (C2).

To systematically investigate the role of miRNAs in ccRCC, we conducted a set of analyses. The study design was illustrated in Supplementary Figure S1. We downloaded the miRNA-seq and clinical datasets from TCGA, including 506 tumor tissues. To better understand the prognostic value of miRNAs in ccRCC, we used univariate Cox regression (Figure 1A and Supplementary Table S1) and multivariate cox regression analysis (Figure 1B and Supplementary Table S2) to analyze survival according to the expression of the associated miRNAs in ccRCC samples from the TCGA database. The results identified 45 most significant miRNAs influencing overall survival (OS) of TCGA-retrieved ccRCC patients, with either being a protective factor (hazard ratio, HR < 1) or being an adverse factor (hazard ratio, HR > 1). These miRNAs were named as independent prognosis-related miRNA (ipr-miRNA) in the present study.

### Clustering by ipr-miRNAs for clear cell renal cell carcinoma

Subsequently, based on the transcriptomic patterns of the ipr-miRNAs, we employed the k-means of unsupervised consensus clustering to classify TCGA-retrieved ccRCC patients. K = 2 was then selected as the optimal cluster number after a comprehensive consideration (Figure 1C, Supplementary Figure S2, and Supplementary Table S3). As shown in Figure 1C, when k = 2, ccRCC patients were classified into 2 subgroups, that is, the C1 and C2 subgroups, which had clear boundaries, suggesting a stable and reliable clustering for the ccRCC patients. Then, the principal component analysis (PCA) and t-distributed stochastic neighbor embedding (t-SNE) analysis were further applied to validate the assignments of the two subtypes, and the results from both methods showed that samples in one subgroup were closer to each other than those in the other subgroup, which suggests the two-dimensional PCA, t-SNE distribution and the two subtypes had similar consistency (Figures 1D,E). To further explore the clinical significance of ccRCC subgroups, we mapped the TCGA-retrieved ccRCC patients to corresponding subgroups and found the survival time between different subgroups showed dramatic difference (Figure 1F). The survival curve revealed that C1 had a dramatically better survival outcome when compared to C2 in overall survival (*p*-value < 0.001) (Figure 1F).

To validate the role of ipr-miRNAs in stratifying ccRCC subgroups, we also employed the k-means of unsupervised consensus clustering to classify an independent GEO-retrieved ccRCC patients (GSE131959) based on the expression of ipr-miRNAs. As shown in Supplementary Figure S3A, GEO-retrieved ccRCC patients were also divided into two subgroups with relatively clear boundaries. When we mapped the GEO-retrieved ccRCC patients to corresponding subgroups, we also found a dramatic discrepancy of survival time between different subgroups (Supplementary Figure S3B). Together, these results suggested the stable and reliable clustering for the ccRCC patients based on the ipr-miRNAs in both train and validation cohorts from TCGA and GEO datasets.

### C1 and C2 subgroups have distinct clinical characteristics and molecular landscapes

**FIGURE 2:**
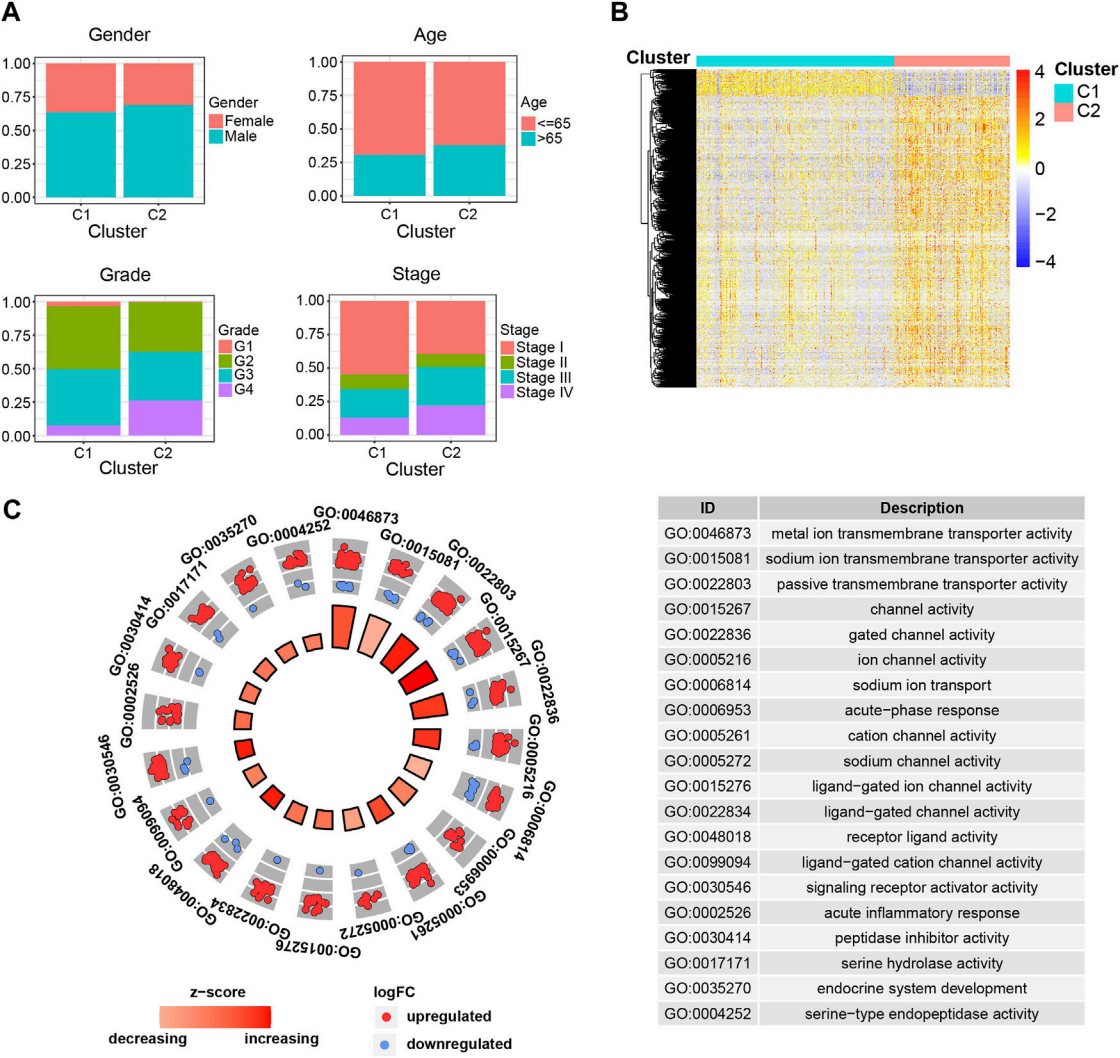
Clinical and molecular differences between the C1 and C2 subgroups. **(A)** Comparison of the clinical characteristics between the indicated subgroups of ccRCC. **(B)** Heatmap shows the differentially expressed genes (DEGs) between the indicated subgroups of ccRCC. **(C)** Functional enrichment of the DEGs.

To further figure out the differences between C1 and C2 subgroups, we investigated the clinical characteristics between the indicated subgroups. We compared the clinical events (age, gender, tumor stage, tumor grade) between the indicated subgroups and found dramatic discrepancies in tumor stage and tumor grade, other than the age and gender, between C1 and C2 subgroups (Figure 2A). The results that patients in C1 subgroup have relatively lower tumor grade and stage not only explained why patients in this subgroup have higher survival probability than patients in C2 subgroup, but also validated the separative capability of the developed stratified system based on the ipr-miRNAs.

Samples derived from different cancer subtypes are often characterized with various molecular features. Thus, we also investigated the molecular differences between the C1 and C2 subgroups (Figures 2B,C). We found a total of 1,650 differentially expressed genes (DEGs), which consist of 1,510 up-regulated and 140 down-regulated genes, between the indicated two subgroups (Figure 2B and Supplementary Table S4). To further examine the biological discrepancies between the indicated two subgroups, functional enrichment analysis was also performed on these DEGs. Consequently, our analysis revealed multiple biological processes that these two subgroups are different, including “metal ion transmembrane transporter activity”, “sodium ion transmembrane transporter activity”, “passive transmembrane transporter activity”, “channel activity”, and “gated channel activity” etc. (Figure 2C). Recent works have demonstrated that cell transmembrane transport activity played vital roles in ccRCC cell survival, which indicated the distinct transmembrane transport activity might be the potential underlying mechanisms account for the dramatic difference in survival between C1 and C2 subgroups (Selvakumar et al., 2014; Liu Y et al., 2015; He and Yang 2019).

Previously, unsupervised cluster analyses of whole genome mRNA expression data have revealed distinct molecular subtypes of ccRCC and the subtypes have been validated being prognostic for clinical outcomes (Brannon et al., 2010; Cancer Genome Atlas Research 2013; Beuselinck et al., 2015; Verbiest et al., 2018). To systematically examine the ccRCC transcriptomic subgroups, we also analyzed the expression profile of independent prognosis-related mRNAs (ipr-mRNAs) across the TCGA-retrieved ccRCCs (Supplementary Table S5). The ipr-mRNAs were then adopted to perform the unsupervised consensus clustering. Similar with the miRNA-derived subgroups, the consensus clustering also showed the optimal performance at K = 2, where ccRCCs were classified into 2 subgroups (C1′ and C2′) (Supplementary Figure S4A). When we mapped the TCGA-retrieved ccRCC patients to corresponding subgroups, we found C1′ had a dramatically better survival outcome compared to C2′ in overall survival (Supplementary Figure S4B). In line with the overall survival rate, patients in C1′ subgroup have relatively lower tumor grade and stage (Supplementary Figure S4C). Notably, when we performed the functional enrichment analysis to enrich the differently expressed mRNA genes between the C1′ and C2′ subgroups, our results also showed these differently expressed mRNA genes were enriched in multiple transmembrane transport related biological processes (Supplementary Figures S4D,E). These results not only revealed the close association between miRNA and mRNA expression patterns in ccRCC patients but also again suggested the important roles of transmembrane transport in ccRCC progression. In addition, when we compared the detailed clinical characteristics of miRNA-derived subgroups and mRNA-derived subgroups, we found miRNA-derived subgroups have relatively better stratified ability than mRNA-derived subgroups as ccRCC patients which specifically enriched in C1 subgroup have relatively lower tumor stage and grade than in C1′ subgroup while patients specifically enriched in C2 subgroup have relatively higher tumor stage and grade than C2’ subgroup (Supplementary Figures S5A–D).

### Ipr-miRNAs play regulatory roles in transmembrane transport

**FIGURE 3:**
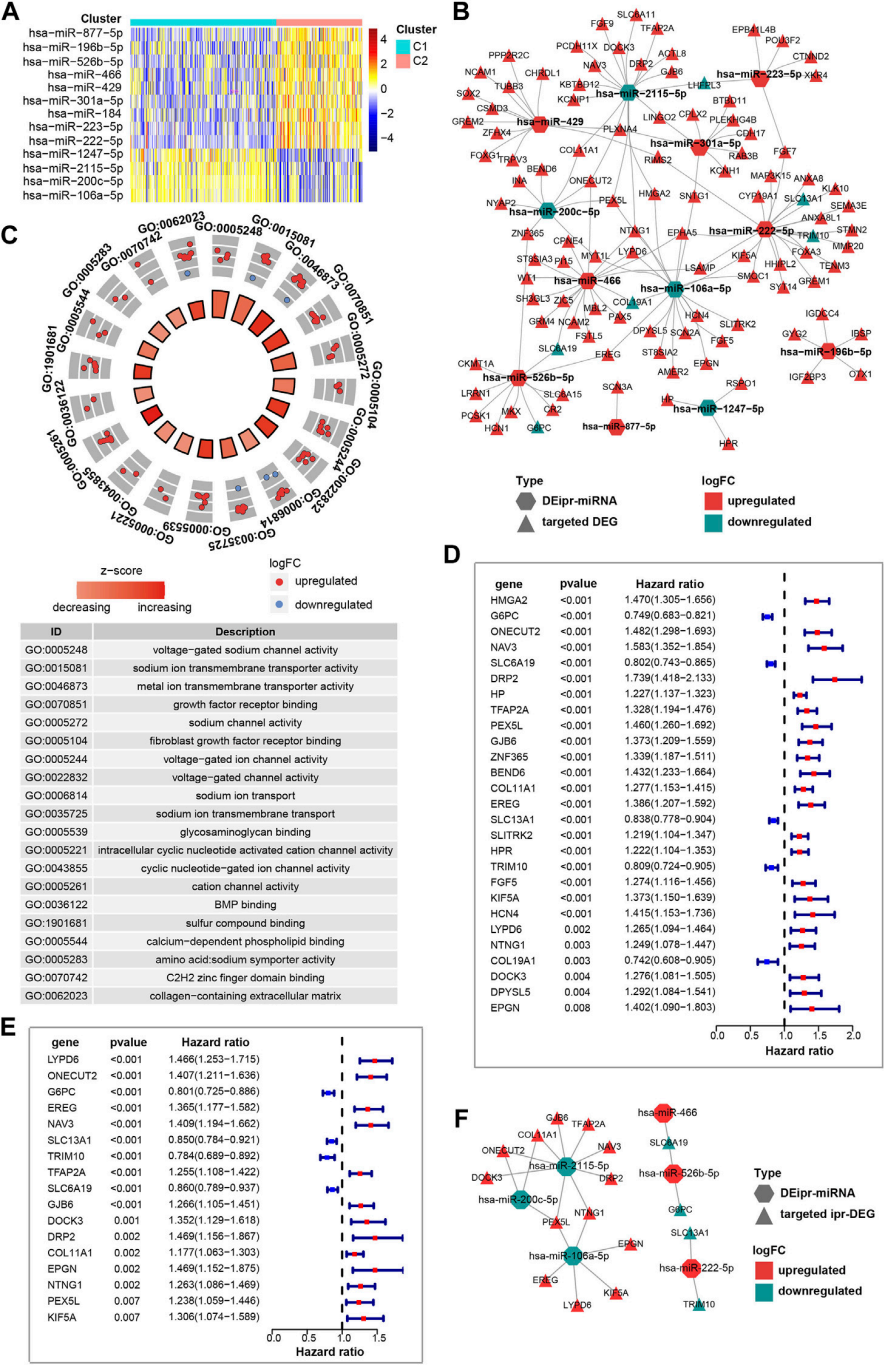
Identification of the prognostic DEipr-miRNAs-regulated DEGs. **(A)** Heatmap shows the differential expressed ipr-miRNAs (DEipr-miRNAs) between the indicated subgroups of ccRCC. **(B)** Regulatory networks of the DEipr-miRNAs and their targeted DEGs. **(C)** Functional enrichment of the DEipr-miRNAs-targeted DEGs. **(D)** Univariate cox regression analysis to identify the prognosis-related DEGs (pr-DEGs). **(E)** Multivariate cox regression analysis to identify the independent prognosis-related DEGs (ipr-DEGs). **(F)** Regulatory networks of the DEipr-miRNAs and reversely expressed ipr-DEGs.

Recent works have shown the transmembrane transport of ion, sodium and other subjects regulate a myriad of tumor-related biological processes. Across various types of cancer, the ion, sodium, etc., channel protein expression and activity are often dysregulated, offering value in stratifying risk and determining the treatment plan (Hu et al., 2021; Fan and Huang 2022). Considering miRNAs play important roles in regulating gene expression, and their dysregulation is closely correlated with cancer initiation and development (He et al., 2020). Thus, in order to understand the role of ipr-miRNAs in the aforementioned biological processes, we started to investigate the interplay between dysregulated miRNAs and the dysregulated genes. Firstly, we investigated the differently expressed miRNAs between C1 and C2 subgroups. A total of 13 miRNAs were identified, which consist of 9 up-regulated and 4 down-regulated miRNAs (Figure 3A). And the Kaplan-Meier analyses validated that the expression of these miRNAs were significantly correlated with patient survivals (Supplementary Figure S6). These miRNAs were named as differently expressed independent prognosis-related miRNAs (DEipr-miRNAs).

Next, to find whether DEipr-miRNAs regulated the expression of the aformentioned DEGs, we predicted the DEipr-miRNA targeted genes by multiple bioinformatic tools and searched if the targeted genes were included in the DEGs list. The TargetScan (http://www.targetscan.org/), miRDB (http://www.mirdb.org/), and miRWalk (http://mirwalk.umm.uni-heidelberg.de/) were used for predicting DEipr-miRNA target genes (Supplementary Figure S7 and Supplementary Table S6). As a result, we found 106 targeted DEGs totally. The network of DEipr-miRNAs and their targeted DEGs are shown in Figure 3B and Supplementary Table S7. Then, to investigated the role of DEipr-miRNAs-targeted DEGs, we performed the functional enrichment analysis. Consequently, our analysis revealed multiple biological processes including “voltage-gated sodium channel activity”, “sodium ion transmembrane transporter activity”, “metal ion transmembrane transporter activity”, “growth factor receptor binding”, and “sodium channel activity” (Figure 3C). The gene ontology results again enriched in the biological processes of transmembrane transport activities suggests DEipr-miRNAs-targeted DEGs mainly functioned in the related biological processes and DEipr-miRNAs might play roles in transmembrane transport activity regulatory network through DEipr-miRNAs-targeted DEGs.

### Identification of prognostic DEipr-miRNAs-regulated DEGs

The canonical role of miRNAs is to initiate decay or block translation of specific target mRNAs and thus negatively regulated targeted gene expression in the cytoplasm (Hill and Tran 2021). For the aforementioned DEipr-miRNAs-targeted DEGs, we also investigated their mRNAs’ subcellular localization. According to the annotation in RNALocate database (https://www.rna-society.org/rnalocate/) (Cui et al., 2022), we found above 95% mRNAs of DEipr-miRNAs-targeted DEGs (101/106, some of them spread in more than one subcellular fraction including membrane, cytosolic, and nuclear fraction) were localized in cytosolic fraction (Supplementary Figure S8, Supplementary Table S8). Thus, to reveal the DEipr-miRNAs-regulated DEGs, we screened out the DEGs which were expressed in an opposite direction to DEipr-miRNAs (Supplementary Table S9).

To better understand the prognostic value of above identified reversely expressed DEGs in ccRCC, we performed univariate cox regression and multivariate cox regression analysis to analyze survival according to the expression of the indicated DEGs in ccRCC samples from the TCGA database. The results identified 17 independent prognosis-related DEGs (ipr-DEGs), including LYPD6, ONECUT2, G6PC, EREG, NAV3, SLC13A1, TRIM10, TFAP2A, SLC6A19, GJB6, DOCK3, DRP2, COL11A1, EPGN, NTNG1, PEX5L, and KIF5A (Figures 3D,E and Supplementary Table S10, Supplementary Table S11). The regulatory network of DEipr-miRNAs and ipr-DEGs were illustrated in the Figure 3F.

### Subcellular localization, expression, clinical features, and KEGG enrichment of ipr-DEGs

Although the subcellular localization and expression patterns of ipr-DEGs at the mRNA levels have been studied, information on the localization and expression of ipr-DEGs at the protein levels remains to be elucidated. Therefore, the immunofluorescence (IF) and immunohistochemistry (IHC) analysis to further identify the expression and subcellular distribution of ipr-DEGs were performed by using the data from Human Protein Atlas database (HPA, https://www.proteinatlas.org/) (Digre and Lindskog 2021). As shown in Supplementary Figure S9, most of the ipr-DEGs (DRP2, EPGN, KIF5A, NAV3, NTNG, ONECUT2 and ALKBH5) showed strong cytoplasmic staining as well as relative weak nuclear staining. Specifically, some proteins (COL11A1, DOCK3, EREG, GJB6, LYPD6) were detected only in the cytoplasm, while TFAP2A signals were found only in the nucleus.

**FIGURE 4:**
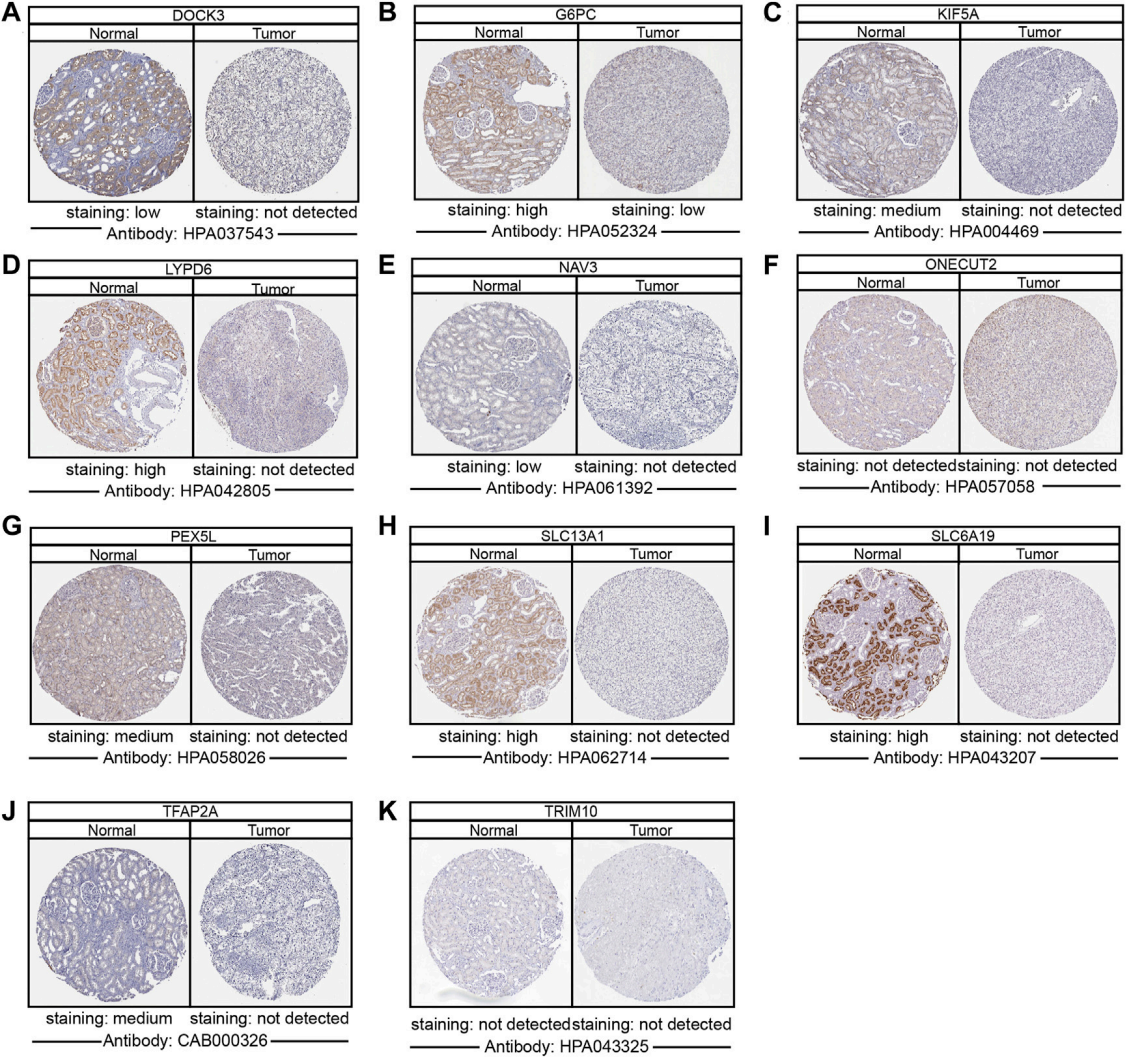
Validation of the expression of DEipr-miRNAs-regulated ipr-DEGs. **(A–K)** The protein expressions of indicated DEipr-miRNAs-regulated ipr-DEGs in ccRCC tumor and normal tissues using clinical specimens from the Human Protein Profiles.

To determine the differentially protein expression of ipr-DEGs, IHC staining images for the ipr-DEGs proteins in ccRCC tissues as well as normal renal tissues were obtained from the HPA database. The results showed that the protein expression levels of DOCK3, G6PC, KIF5A, LYPD6, NAV3, PEX5L, SLC13A1, SLC6A19, and TFAP2A were higher in normal renal tissues than that in ccRCC tissues, while the protein expression levels of ONECUT2 and TRIM10 were not detected (Figure 4). The rest of proteins, including COL11A1, DRP2, EPGN, EREG, GJB6, and NTNG1 were not available in HPA database. Consistent with the above results, transcriptional results from TCGA and ICGC databases showed that DOCK3, KIF5A, LYPD6, PEX5L, SLC13A1, SLC6A19, and TFAP2A were dramatically down-regulated in ccRCC and RCC tumor samples than normal samples (Supplementary Figures S10A,B). Interestingly, we also investigated the copy number variations of the indicated genes in ccRCC tumor and normal samples. The copy number variations resulted from TCGA revealed that little changes in the DNA levels were happened for the ipr-DEGs excluding DOCK3 (Supplementary Figure S10C). Together, these results indicated that the downregulation of DOCK3 might occurred in the gene levels, while the downregulation of KIF5A, LYPD6, PEX5L, SLC13A1, SLC6A19, and TFAP2A might occurred in the post-transcriptional levels directed by DEipr-miRNAs.

We further validated the correlation between the expression of ipr-DEGs and overall survivals in ccRCC to explore the clinical significance of ipr-DEGs’ expression. As shown in Supplementary Figure S11, Kaplan-Meier analyses revealed that, except LYPD6 and EPGN, the expression of ipr-DEGs were all significantly correlated with ccRCC patient survivals.

To further investigate the detailed functional mechanisms of the identified ipr-DEGs, we uploaded the indicated ipr-DEGs to the online tool KEGG pathway analysis (https://www.genome.jp/kegg/tool/map_pathway2.html). KEGG analysis enriched SLC13A1, SLC6A19, and PEX5L in cellular biosynthesis and metabolism related pathways through regulating the transportation of Na^+^, amino acid, and proteins. TFAP2A and KIF5A were enriched in endocytosis processes of multiple cell growth factors (Supplementary Figure S12A). Cellular biosynthesis, metabolism, and the function of growth factor are widely recognized as important regulators of ccRCC tumor proliferation and survival (Wettersten et al., 2017; Jonasch et al., 2021). Thus, the enrichment of KIF5A, PEX5L, SLC13A1, SLC6A19, and TFAP2A in the aforementioned pathways indicates these ipr-DEGs are functioned through regulating the activity of transmembrane transport, biosynthesis, metabolism, and endosome and peroxisome systems to regulate the development of ccRCC and the dysregulation of these ipr-miRNAs regulated ipr-DEGs drives the generation of ccRCC samples with distinct clinical and biological characteristics (Supplementary Figure S12B). Together, these results revealed an ipr-miRNA centered ipr-DEGs regulatory network involved in ccRCC prognosis.

## Discussion

The Consensus Molecular Subtypes (CMSs) have implications for our understanding of tumor heterogeneity and the prognosis of patients (Guo et al., 2021; Zhong et al., 2021). So far, the classification has been widely based on the use of messenger RNAs (mRNAs), although miRNAs have been shown to play vital roles in tumor heterogeneity and biological differences between CMSs (Lu et al., 2005; Adam et al., 2022). In contrast to mRNAs, miRNAs have a smaller size and increased stability, facilitating their detection and thus became an important matter for biomarker researchers. In the present study, we collected 506 TCGA-retrieved ccRCC patients and presented a comprehensive transcriptomic and clinical-related analysis of miRNAs. The ccRCC patients were classified into two subgroups (C1 and C2) exhibiting different biological properties based on transcriptomic prognosis-related miRNA profiling. The patients in C1 subgroup are characterized with relatively lower tumor stage, grade, and a better overall survival while the patients in C2 subgroup are characterized with relatively higher tumor stage, grade, and a poor overall survival.

In addition to the clinical differences between the C1 and C2 subgroups, we also found discriminating features in the expression of genes, which were further enriched in the biological processes related to transmembrane transporter activities for transport ion, sodium, and other subjects. Cell membrane transport function plays vital roles in many aspects of tumorigenesis. For example, our previous work delineated that regulating the transport of H^+^ through carbonic anhydrase Ⅸ would dramatically affect the proliferation and metastasis of tongue squamous carcinomas (Shen et al., 2021). Selvakumar P et al. found that knockdown of the von Hippel-Lindau (VHL) tumor suppressor gene in renal cell carcinoma (RCC) cell lines would disturb the expression of Na^+^ and K^+^ transported proteins which is associated with RCC initiation and progression (Selvakumar et al., 2014). Wu YY et al. showed the activity of ligand-gated Ca2^+^ channel was significantly associated with primary human RCC Fuhrman grades and histopathological subtypes (Wu et al., 2018). These reports not only supported the close relationship of transmembrane transporter activities with ccRCC initiation and progression, but also indicated the important roles of the transmembrane transporter activities in regulating different ccRCC subtypes.

Across various types of cancer, the ion, sodium, etc., channel protein expression and activity are often dysregulated and offered value in stratifying risk and determining the treatment plan (Hu et al., 2021; Fan and Huang 2022). In the present study, we identified and validated the regulation of KIF5A, PEX5L, SLC13A1, SLC6A19, and TFAP2A were occurred in the post-transcriptional levels directed by DEipr-miRNAs. Among these validated ipr-DEGs, KIF5A is reported to regulate the transport of endosomal vesicles and control autophagic flux, which might regulate renal tumorigenesis through mediating HIF2α degradation (Schmidt et al., 2009; Liu X et al., 2015; Liu et al., 2021). PEX5L, also named as TRIP8b, is suggested to function in vesicle transport in mouse pituitary tumor AtT20 cells (Chen et al., 2001). Genetic variation of PEX5L is closely related to the function of peroxisomes and non-small cell lung cancer survival (Chen et al., 2022). SLC13A1 and SLC6A19 are identified as typical and novel renal amino acid cotransporter which imports a broad range of neutral amino acids, ion, and Na^+^ (Verrey et al., 2005; Markovich and Aronson 2007). The dysregulation of SLC13A1 and SLC6A19 are reported to affect various type of tumor progression including ccRCC (Horinouchi et al., 2010; Bogatikov et al., 2012; Choudhury et al., 2015). TFAP2A served as typical epigenetic marker for ccRCC and regulates potassium (K^+^) channel tetramerization domain containing 15a and 15b (Kctd15a and 15b) (Dalgin et al., 2008; Chambers et al., 2020). The interplay between TFAP2A and miRNAs can manifest the survival of ccRCC patients (Qin et al., 2019). The involvement of these ipr-DEGs in transmembrane transporter regulation is closely associated with tumorigenesis and progression, which support the undiscovered functions in transmembrane transporter and tumor-related roles of their upstream regulators DEipr-miRNAs in ccRCC.

miRNAs are small nucleotides with wide regulatory functions including initiating decay or blocking translation of specific target mRNAs. Thus, we investigated the correlation of the dysregulated prognostic miRNAs (DEipr-miRNAs) and the aforementioned ipr-DEGs. After targeting prediction, regulatory network analysis, hub module investigation (including CNV, expression, and subcellular localization analysis), and prognostic validation, we eventually found miR-2115–5p, miR-200c-5p, miR-106a-5p, miR-466, and miR-222–5p are the upstream regulators of the aforementioned ipr-DEGs. Among these miRNAs, miR-106a-5p has been proved to be a tumor suppressor by targeting VEGFA in RCC (Ma et al., 2020). In our analysis, we also found miR-106a-5p is highly expressed in C1 subgroup, which have relatively lower tumor grade, stage, and survival probability. miR-222–5p has been reported to highly expressed in ccRCC tumor cells and repress the express of TRIM2 and thus promote the progression and prognosis of metastatic ccRCC (Wei et al., 2020). Correspondingly, in our study, we found miR-222–5p is highly expressed in C2 subgroup, which have relatively higher tumor grade, stage, and survival probability. Although the other miRNAs have not been investigated in ccRCC yet, previous studies have reported that the expression of miR-200c-5p (Li et al., 2017) and miR-466 (Colden et al., 2017) were associated with tumorigenesis and progression in various tumor types, which support their further identification and exploring in ccRCC. For miR-2115–5p, although there has been no direct evidence demonstrating that miR-2115–5p plays a role in tumor progression yet, our results implicate the potential relevance of miR-2115–5p in ccRCC.

The classification based on prognostic miRNA expressions in our study will be helpful for biological function, pre-clinical precision meditation and target-therapy research. The patients from different subgroups showed distinct molecular landscapes and clinical outcomes, which suggests potential strategies of more efficient clinical management for patients in different subgroups. Covering more ccRCCs in the future works will offer a relatively more comprehensive characterization. In addition, further efforts are needed to validate the role of aforementioned miRNAs in regulating the predicted pathways and ccRCC progression in the future works. Up to date, our study provided a comprehensive picture of molecular alterations in ccRCC from the aspect of miRNA, which contributes much to the understanding of ccRCC. The subgroups based on miRNA expression exhibited high consistence in both PCA and t-SNE methods, which indicates that miRNA expression is sufficient to catch the major biological discrepancies among different subgroups.

## Conclusion

In summary, our study recapitulated molecular and clinical features of clear cell renal cell carcinoma patients through miRNA transcriptome, unveiled potential targets served as effective biomarkers in multiple layers, and would accelerate the development of diagnosis and prognosis for clear cell renal cell carcinoma patients.

## Data Availability

The original contributions presented in the study are included in the article/Supplementary Material, further inquiries can be directed to the corresponding author.
