# Supplementary material for: Characterizing the molecular heterogeneity of clear cell renal cell carcinoma subgroups classified by miRNA expression profile
Source: Front Mol Biosci. 2022 Aug 26;9:967934. doi: 10.3389/fmolb.2022.967934 (PMC9459094; doi:10.3389/fmolb.2022.967934)
Supplement: Supplementary file 1 [file DataSheet1.pdf]

Supplementary Figures

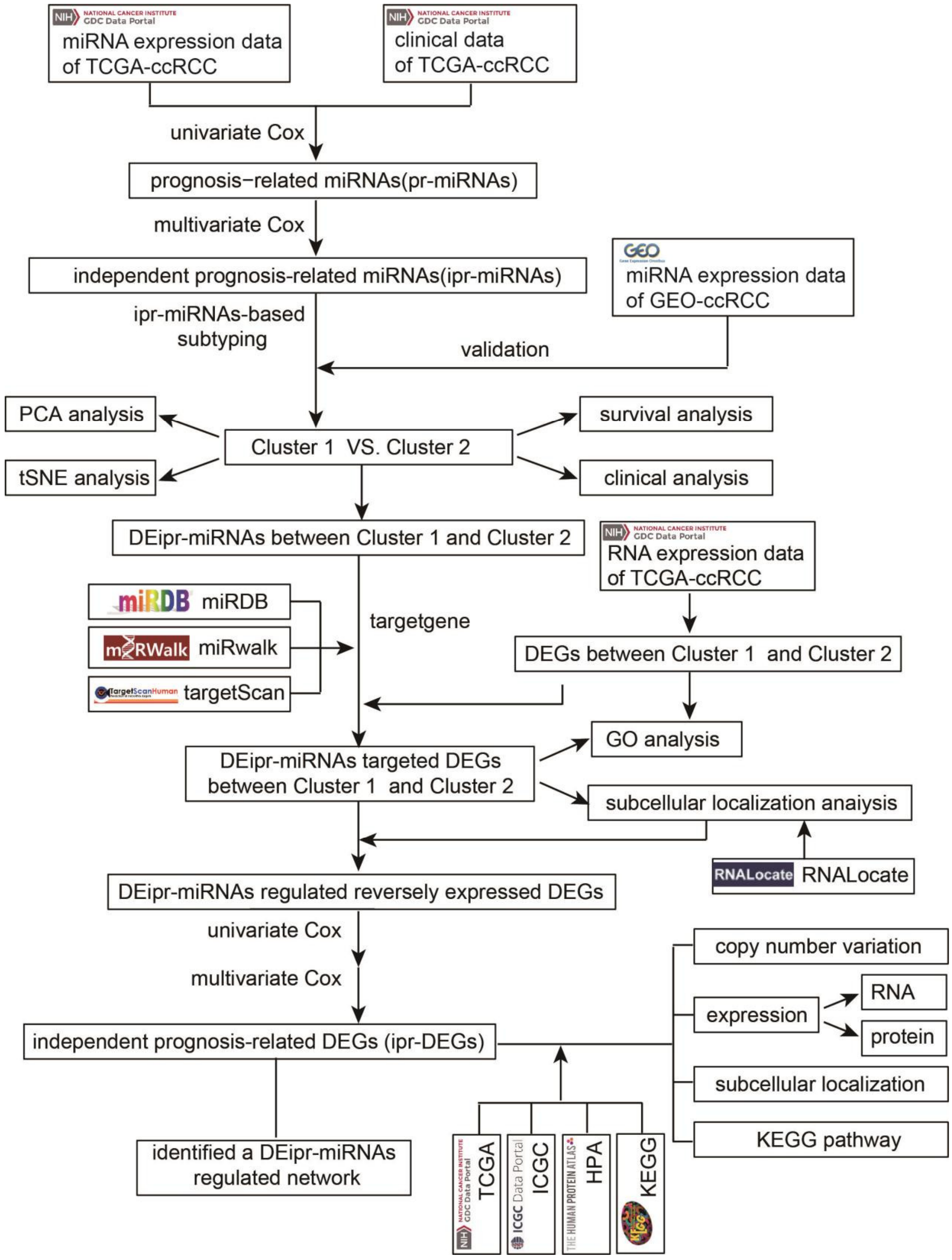

Figure S1. Schematic view of the procedures for data collection and analyses in ccRCC.

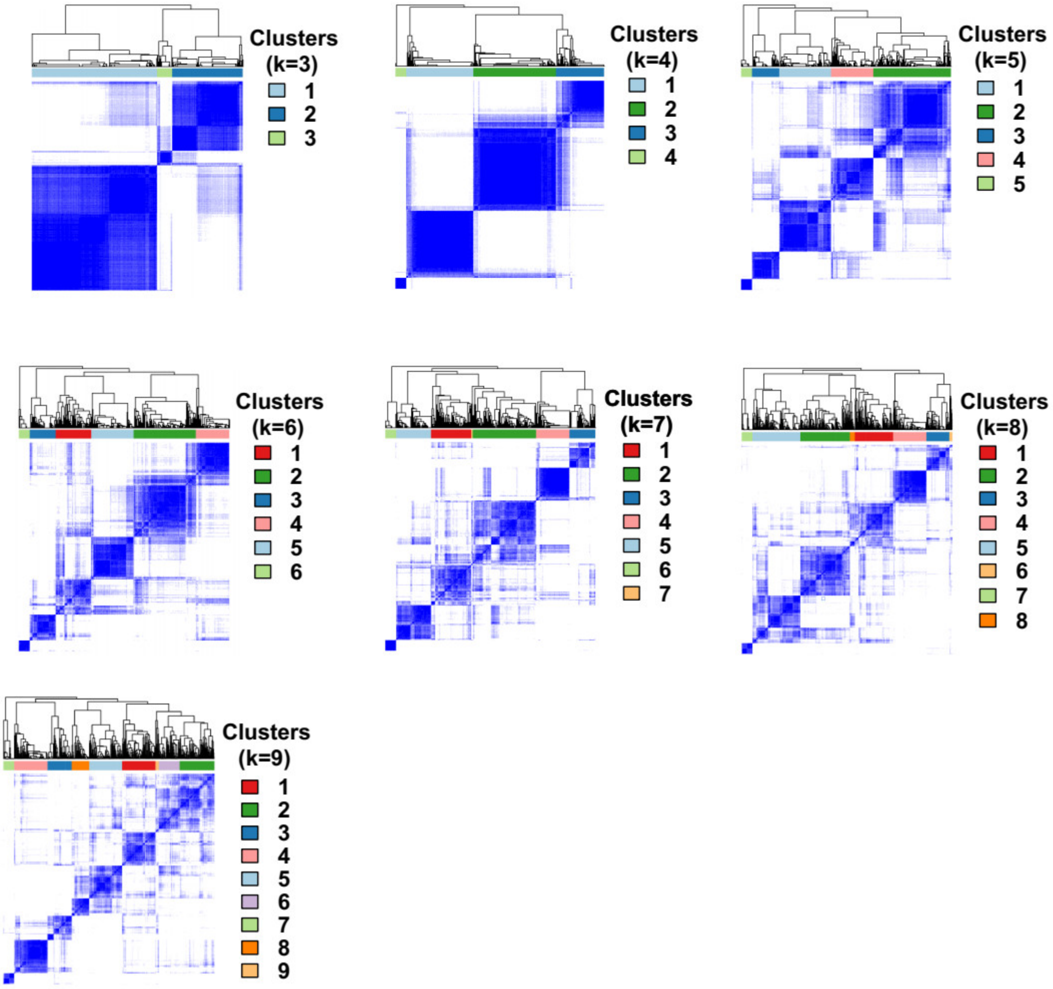

Figure S2. Consensus matrix heatmap when k = 3-9. Related to Figure 1C.

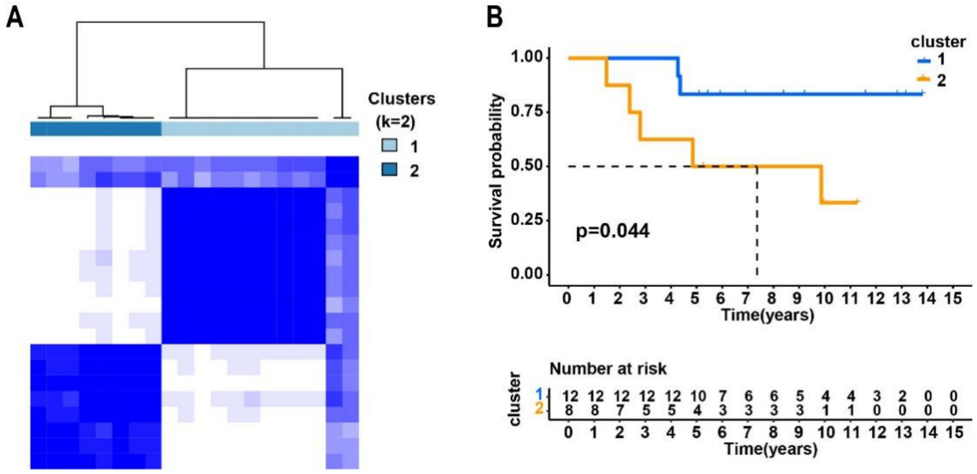

Figure S3. Consensus clustering based on the independent prognosis-related miRNAs for GEO-retrieved ccRCC patients. **(A)** Consensus matrix heatmap (n=20). **(B)** Kaplan-Meier plot analysis for the indicated GEO-retrieved ccRCC patients distributed in Cluster1 and Cluster2.

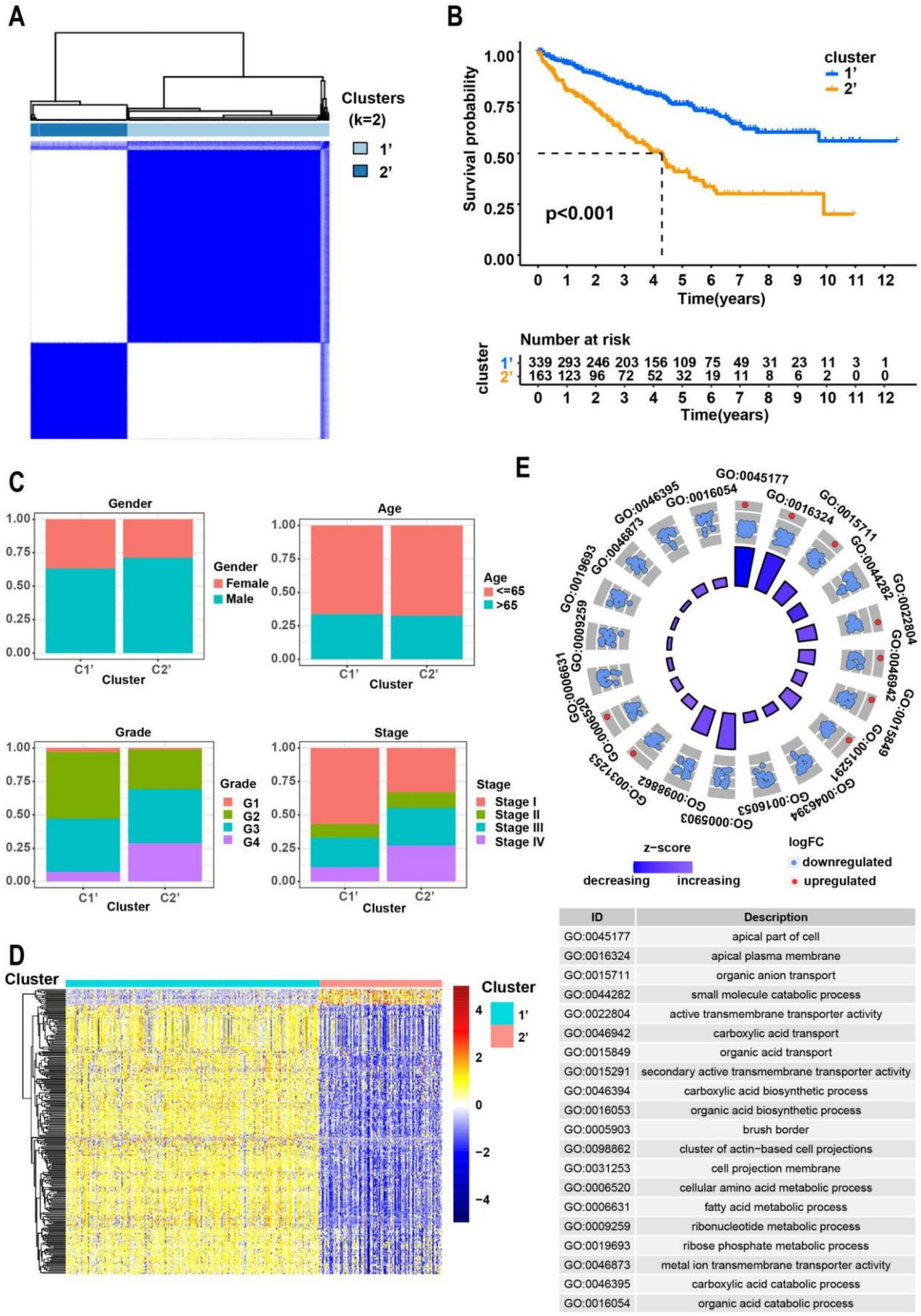

Figure S4. Consensus clustering based on the independent prognosis-related mRNAs for TCGA-retrieved ccRCC patients. **(A)** Consensus matrix heatmap. **(B)** Kaplan-Meier plot analysis for the indicated TCGA-retrieved ccRCC patients distributed in Cluster1' and Cluster2'. **(C)** Comparison of the clinical characteristics between the indicated subgroups of ccRCC. **(D)** Heatmap shows the differential expressed mRNA genes between the indicated subgroups of ccRCC. **(E)** Functional enrichment of the differential expressed mRNA genes.

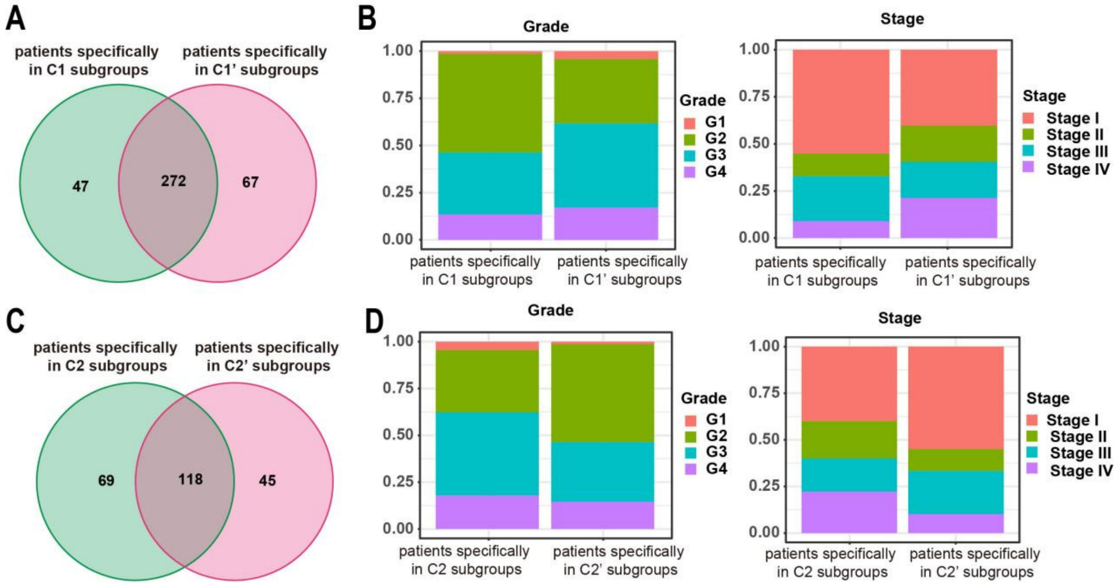

Figure S5. Comparation of the clinical characteristics between miRNA-derived subgroups and mRNA-derived subgroups. **(A)** Venn diagram of ccRCC patients in C1 and C1' subgroups. **(B)** Comparison of the clinical characteristics between patients specifically enriched in C1 and C1' subgroups. **(C)** Venn diagram of ccRCC patients in C2 and C2' subgroups. **(D)** Comparison of the clinical characteristics between patients specifically enriched in C2 and C2' subgroups.

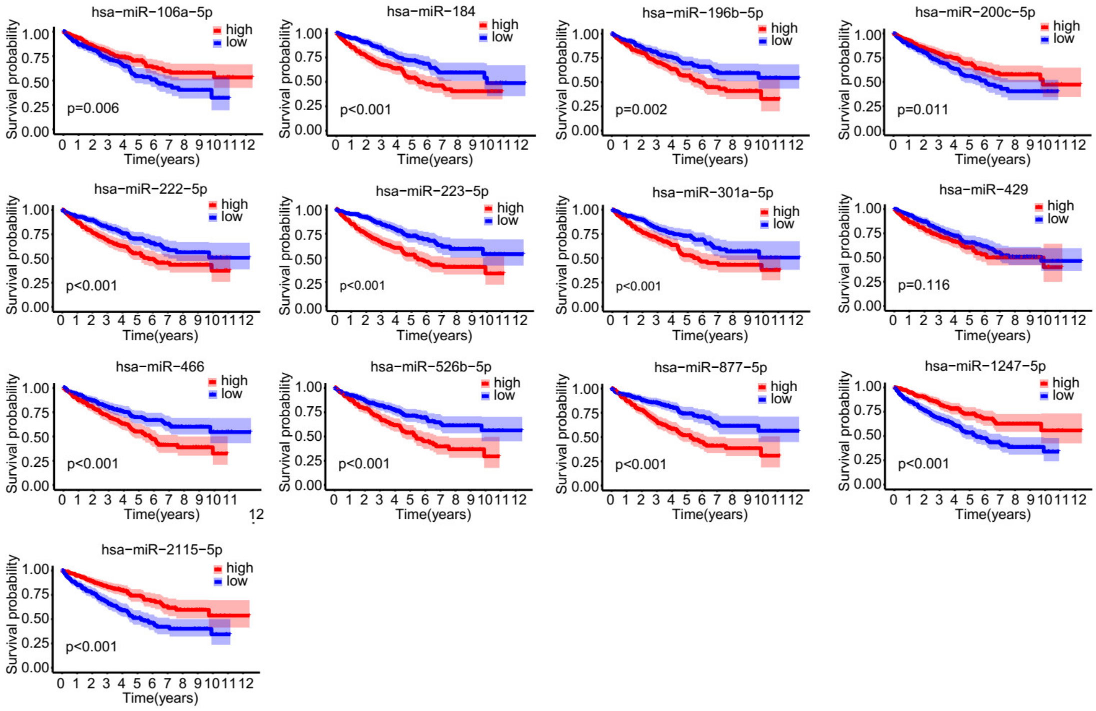

Figure S6. Kaplan-Meier plot analysis for the indicated miRNAs in the TCGA-retrieved ccRCC patients.

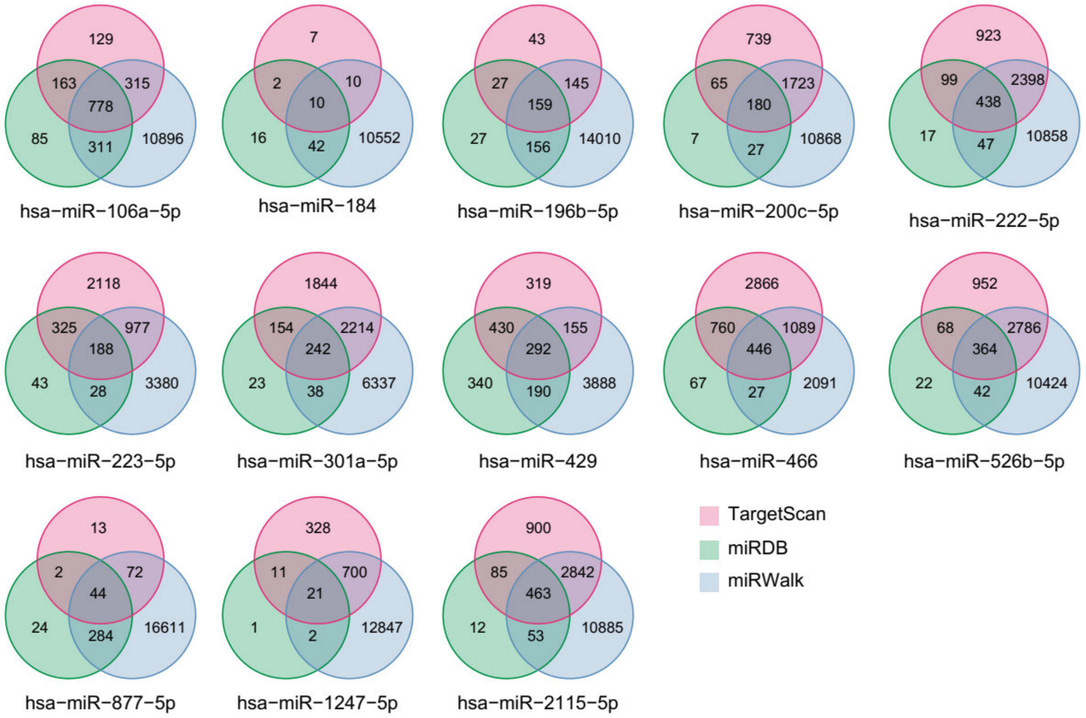

Figure S7. Targets prediction of the DEipr-miRNAs by TargeScan, miRDB and miRWalk.

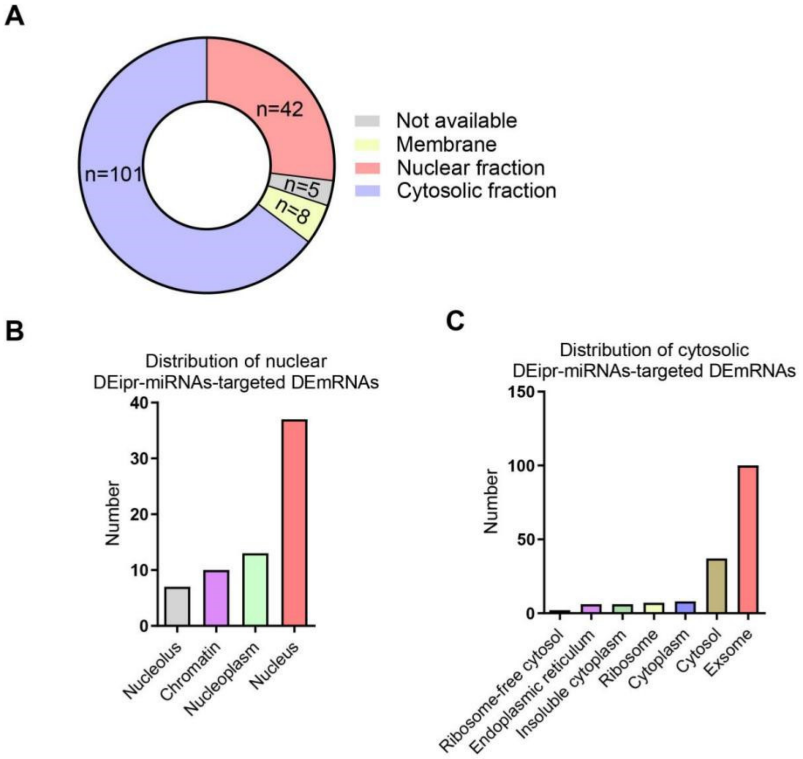

Figure S8. Subcellular distribution of DEipr-miRNA-targeted DEGs' mRNAs. **(A)** Annotated subcellular distribution of DEipr-miRNA targeted dysregulated mRNAs by RNALocate database. **(B)** Annotated cytosolic distribution of DEipr-miRNA targeted DEGs by RNALocate database. **(C)** Annotated cytosolic distribution of DEipr-miRNA targeted DEGs by RNALocate database.

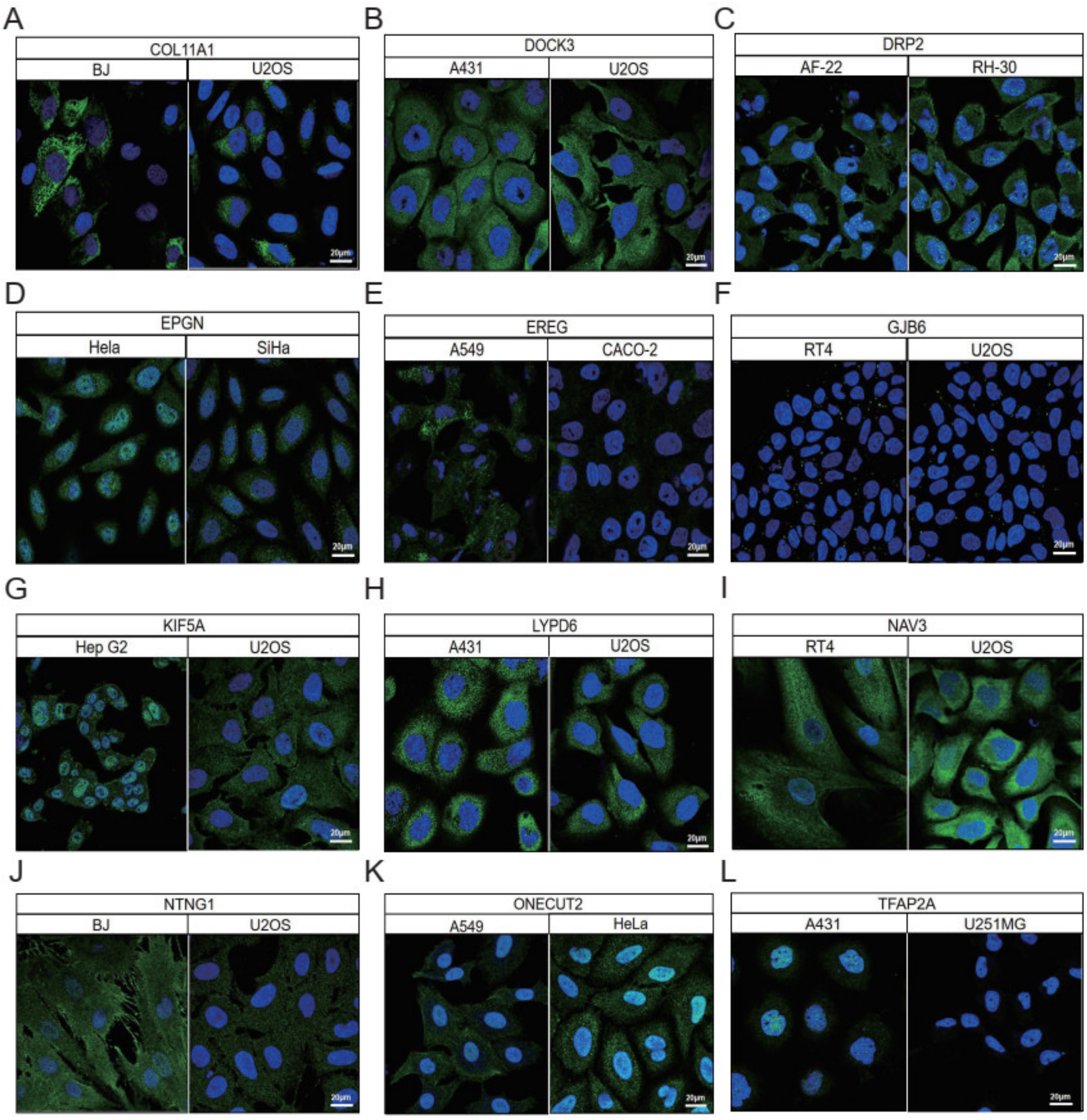

Figure S9. Subcellular localization of the DEipr-miRNAs-regulated iprDEGs' protein in the indicated cell lines. Scale bar= 20μm.

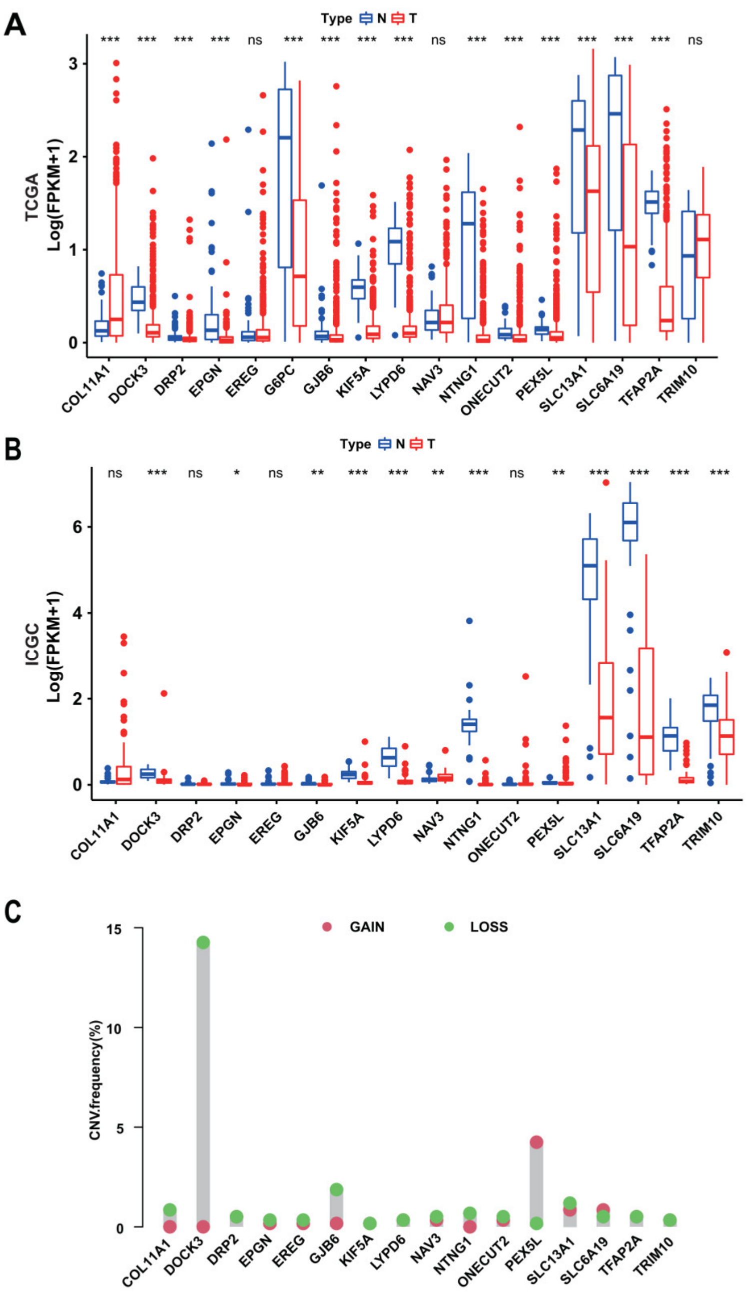

Figure S10. The copy number variation and RNA expression level changes of the indicated DEipr-miRNAs-regulated iprDEGs. **(A)** The RNA expressions of DEipr-miRNAs-regulated iprDEGs in TCGA-retrieved ccRCC tumor and normal samples. T, tumor= 535, N, normal= 72. **(B)** The RNA expressions of DEipr-miRNAs-regulated iprDEGs in ICGC-retrieved RCC tumor and normal samples. T, tumor= 91, N, normal= 45. **(C)** The copy number variation of the indicated DEipr-miRNAs-regulated iprDEGs.

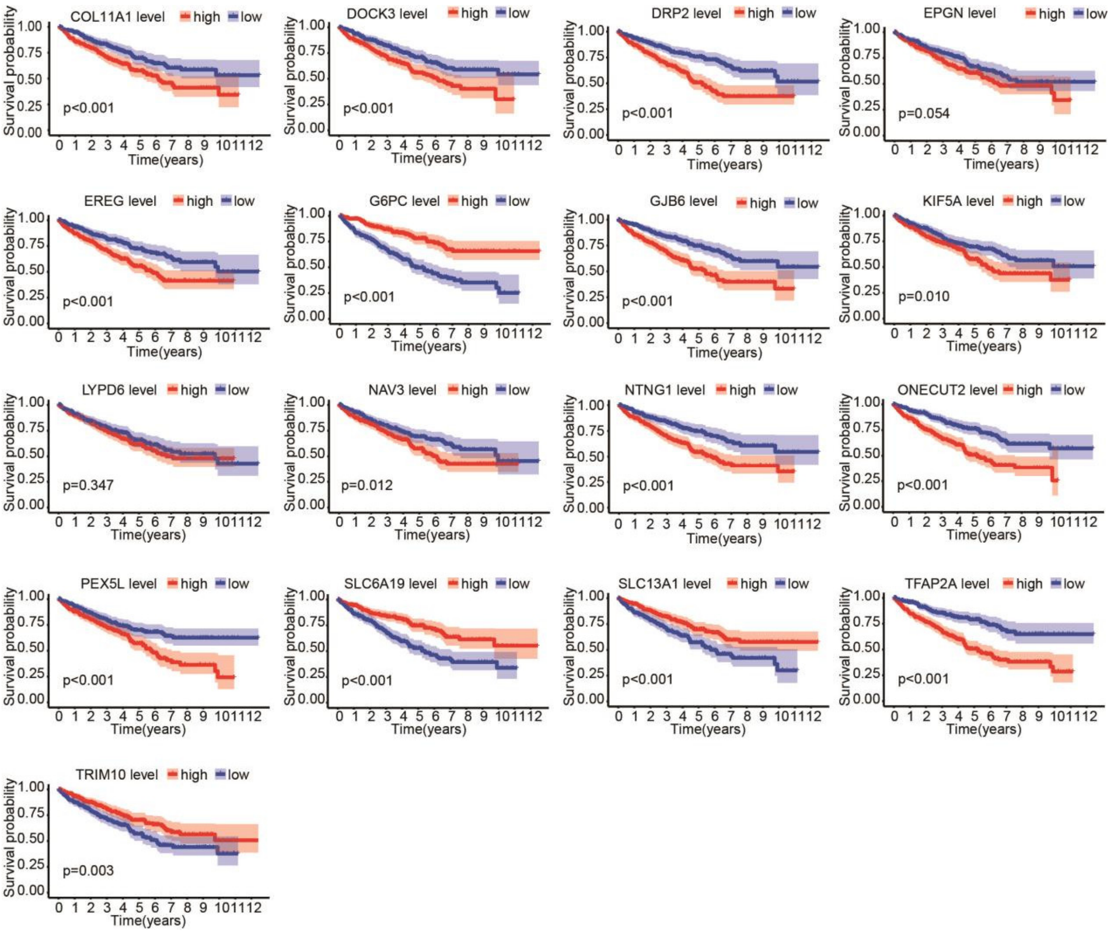

Figure S11. Kaplan-Meier plot analysis for the indicated DEipr-miRNAs-regulated iprDEGs.

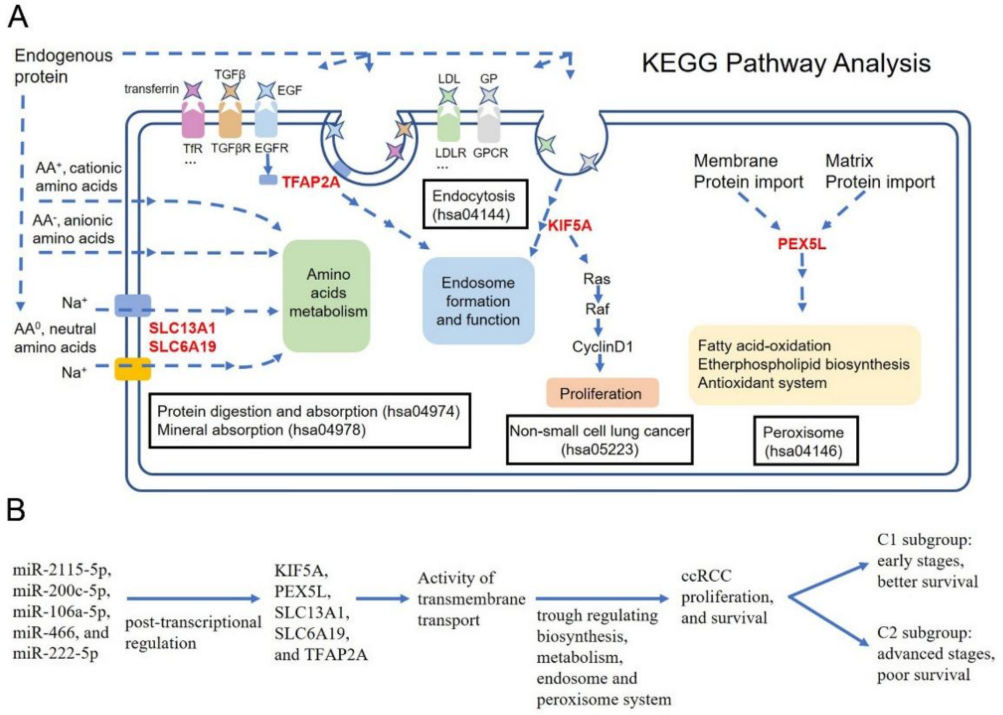

Figure S12. Enrichment of the indicated ipr-DEGs. **(A)** KEGG pathway analysis of the indicated iprDEGs. **(B)** A schematic illustration of the regulatory axis by which ipr-miRNAs regulated ipr-DEGs directed ccRCC tumor biological behavior and characteristics.
